# Supplementary material for: Characterization of Co-Formulated High-Concentration Broadly Neutralizing Anti-HIV-1 Monoclonal Antibodies for Subcutaneous Administration
Source: Antibodies (Basel). 2020 Jul 29;9(3):36. doi: 10.3390/antib9030036 (PMC7551838; doi:10.3390/antib9030036)
Supplement: Supplementary file 1 [file antibodies-09-00036-s001.pdf]

Supplementary Information (Sharma et al.)

Table S1. Precision and accuracy of the reverse phase chromatography method during analysis of co-formulated antibodies. Intra- and Inter- assay precision of (A) total peak area (10-1074-LS and 3BNC117-LS peaks), (B) 10-1074-LS-specific peak area and (C) 3BNC117-LS-specific peak area from six replicate runs of co-formulated samples analyzed by reverse phase chromatography. (D) Accuracy determination from percent recovery of a range of co-formulated samples analyzed on three different days (day 1, 2 and 3).

(A)

**Conc. (2.5 + 2.5 µg)**	**Total Peak Area**		
**Replicates**	**Day 1**	**Day 2**	**Day 3**
1	5059	5048	5067
2	5071	5041	5065
3	5066	5029	5070
4	5060	5037	5058
5	5075	5066	5060
6	5049	5050	5077
Intra-assay Precision (%RSD)	0.2%	0.3%	0.1%
Inter-assay Precision (% RSD)	0.3%		

(B)

**Conc. (2.5 µg)**	**10-1074-LS Peak Area**		
**Replicates**	**Day 1**	**Day 2**	**Day 3**
1	2390	2396	2408
2	2404	2397	2408
3	2407	2399	2411
4	2388	2398	2402
5	2409	2403	2402
6	2404	2383	2406
Intra-assay Precision (%RSD)	0.4%	0.3%	0.1%
Inter-assay Precision (% RSD)	0.3%		

(C)

**Conc. (2.5 µg)**	**3BNC117-LS Peak Area**		
**Replicates**	**Day 1**	**Day 2**	**Day 3**
1	2547	2537	2536
2	2546	2538	2536
3	2545	2530	2536
4	2552	2531	2536
5	2547	2544	2543
6	2540	2540	2540

Intra-assay Precision (%RSD)	0.2%	0.2%	0.1%
Inter-assay Precision (% RSD)	0.2%		

(D)

Concentration (μg)	% Recovery		
	**Day 1**	**Day 2**	**Day 3**
1	102.0%	102.2%	101.3%
2	99.5%	99.8%	99.3%
4	99.8%	99.6%	99.8%
5	99.8%	99.6%	100.1%
6	100.0%	100.0%	100.2%
8	100.2%	100.2%	99.9%

Table S2. Precision and accuracy of the cation-exchange chromatography method during analysis of co-formulated antibodies. Intra- and Inter- assay precision of (A) total peak area (10-1074-LS and 3BNC117-LS peaks), (B) 10-1074-LS-specific peak area and (C) 3BNC117-LS-specific peak area from six replicate runs of co-formulated samples analyzed by cation-exchange chromatography. (D) Accuracy determination from percent recovery of a range of co-formulated samples analyzed on three different days (day 1, 2 and 3) for 6 different concentrations (50, 80, 100, 120, 200 and 300 μg).

(A)

**Conc. (50 + 50 μg)**	**Total Peak Area**		
**Replicates**	**Day 1**	**Day 2**	**Day 3**
1	14583	14371	15365
2	15036	14780	15269
3	14913	14539	15682
4	14767	14985	15014
5	15000	15263	15308
6	14865	14884	15272
Intra-assay Precision (% RSD)	1.1%	2.2%	1.4%
Inter-assay Precision (% RSD)	2.2%		

(B)

**Conc. (50 μg)**	**10-1074-LS Peak Area**		
**Replicates**	**Day 1**	**Day 2**	**Day 3**
1	6903	6804	7168
2	7103	6951	7149
3	7098	6941	7313
4	7019	6958	6976
5	7033	7221	7181
6	6994	7115	7179
Intra-assay Precision (% RSD)	1.1%	2.1%	1.5%
Inter-assay Precision (% RSD)	1.8%		

(C)

**Conc. (50 μg)**	**3BNC117-LS Peak Area**		
**Replicates**	**Day 1**	**Day 2**	**Day 3**
1	7680	7568	8197
2	7933	7828	8119
3	7815	7598	8369
4	7747	8027	8038

5	7967	8042	8038
6	7871	7769	8093
Intra-assay Precision (% RSD)	1.4%	2.6%	1.5%
Inter-assay Precision (% RSD)	2.7%		

(D)

**Concentration (µg)**	**% Recovery**		
	**Day 1**	**Day 2**	**Day 3**
50	104.2%	102.7%	99.9%
80	98.7%	98.8%	98.1%
100	98.9%	98.7%	100.6%
120	99.9%	100.0%	101.4%
200	99.7%	100.6%	99.8%
300	100.3%	99.9%	100.0%

Table S3. Precision and accuracy of the size-exclusion chromatography method during analysis of co-formulated antibodies. Intra- and Inter- assay precision of (A) main peak area (10-1074-LS and 3BNC117-LS monomer), (B) High Molecular Weight (HMW) peak area and (C) Low Molecular Weight (LMW) peak area from six replicate runs of co-formulated samples analyzed by size-exclusion chromatography. (D) Accuracy determination from percent recovery of a range of co-formulated samples analyzed on three different days (day 1, 2 and 3) for 6 different concentrations (50, 80, 100, 120, 200 and 300 µg).

(A)

**Conc. (50+50 µg)**	**Main Peak Area**		
**Replicates**	**Day 1**	**Day 2**	**Day 3**
1	96.28	95.96	95.90
2	96.27	95.96	95.88
3	96.27	95.99	95.92
4	96.27	96.03	95.87
5	96.26	96.00	95.82
6	96.22	96.00	95.85
Intra-assay Precision (%RSD)	0.0%	0.0%	0.0%
Inter-assay Precision (%RSD)	0.2%		

(B)

**Conc. (50+50 µg)**	**HMW Peak Area**		
**Replicates**	**Day 1**	**Day 2**	**Day 3**
1	3.64	3.97	4.02
2	3.66	3.95	4.03
3	3.66	3.93	4.02
4	3.66	3.91	4.07
5	3.66	3.93	4.09
6	3.70	3.93	4.07
Intra-assay Precision (%RSD)	0.5%	0.5%	0.7%
Inter-assay Precision (%RSD)	4.3%		

(C)

**Conc. (50+50 µg)**	**LMW Peak Area**		
**Replicates**	**Day 1**	**Replicates**	**Day 1**
1	0.08	0.07	0.08
2	0.06	0.09	0.09
3	0.07	0.08	0.06
4	0.07	0.06	0.06
5	0.07	0.07	0.09
6	0.08	0.07	0.07
Intra-assay Precision (%RSD)	10.5%	14.1%	18.4%
Inter-assay Precision (%RSD)	14.0%		

(D)

**Concentration (µg)**	**% Recovery**		
	**Day 1**	**Day 2**	**Day 3**
50	102.2%	100.7%	100.0%
80	98.6%	99.0%	98.7%
100	99.8%	100.1%	100.2%
120	100.3%	100.6%	100.6%
200	99.7%	99.8%	100.3%
300	100.1%	100.1%	99.8%

Table S4. Summary of 28-day stability testing results of co-formulated antibodies, 3BNC117-LS and 10-1074-LS (each at 75 mg/mL), evaluated at 0, 1, 2, 3, and 4 weeks, after incubation at accelerated conditions of 25 ±2°C/RH 60% ± 5%. HMW = High Molecular Weight; PDI = Polydispersity Index; P/mL = Particles/mL.

**Test Attributes**		**Weeks**				
		**0**	**1**	**2**	**3**	**4**
**pH**		5.65	5.62	5.64	5.61	5.61
**A280 (mg/mL)**		142	145	144	140	146
**Viscosity (cP)**		10.70	11.81	11.64	11.02	12.51
**Osmolality (mOsm/Kg)**		345	336	345	333	345
**SE-HPLC**	HMW (%)	2.98	3.35	3.14	3.69	3.77
	Main Peak (%)	96.90	96.57	96.75	96.16	96.07
**CEX-HPLC 3BNC117-LS**	Main Peak (%)	48.78	48.62	49.66	49.03	49.69
	Pre-Main Peaks (%)	47.68	46.00	45.48	45.19	45.92
	Post-Main Peaks (%)	3.54	5.38	4.86	5.78	4.39
**CEX-HPLC 10-1074-LS**	Main Peak (%)	32.68	34.86	34.99	34.41	33.39
	Pre-Main Peaks (%)	61.64	59.42	59.02	60.09	61.47
	Post-Main Peaks (%)	5.68	5.72	5.99	5.50	5.14
**RP-HPLC**	3BNC117-LS (mg/mL)	68.05	70.31	64.16	69.19	66.94
	10-1074-LS (mg/mL)	76.70	80.17	72.60	79.26	77.10
**DLS**	Z-Average (d.nm)	10.25	10.25	10.28	10.26	10.33
	PDI	0.18	0.18	0.18	0.18	0.18
**FlowCAM**	2-10 µm (P/mL)	191	658	194	146	276
	10-25 µm (P/mL)	31	69	28	15	61
	25-50 µm (P/mL)	15	38	0	0	8

Table S5. Summary of 28-day stability testing results of co-formulated antibodies, 3BNC117-LS and 10-1074-LS, evaluated at 0, 1, 2, 3, and 4 weeks, after incubation at stressed conditions of 40 ± 2°C/75 ± 5 % RH. HMW = High Molecular Weight; PDI = Polydispersity Index; P/mL = Particles/mL.

**Test Attributes**		**Weeks**				
		**0**	**1**	**2**	**3**	**4**
**pH**		5.65	5.66	5.59	5.69	5.61
**A280 (mg/mL)**		142	148	147	144	149
**Viscosity (cP)**		10.70	11.19	11.39	12.42	12.65
**Osmolality (mOsm/Kg)**		345	348	359	338	359
**SE-HPLC**	HMW (%)	2.98	3.55	3.69	4.29	4.52
	Main Peak (%)	96.90	96.20	95.99	95.23	94.92
**CEX-HPLC 3BNC117-LS**	Main Peak (%)	48.78	49.53	47.85	49.75	48.28
	Pre-Main Peaks (%)	47.68	45.76	45.73	46.04	47.34
	Post-Main Peaks (%)	3.54	4.71	6.42	4.21	4.38
**CEX-HPLC 10-1074-LS**	Main Peak (%)	32.68	29.17	26.34	19.85	18.68
	Pre-Main Peaks (%)	61.64	65.05	68.88	76.18	77.66
	Post-Main Peaks (%)	5.68	5.78	4.79	3.98	3.66
**RP-HPLC**	3BNC117-LS (mg/mL)	68.05	68.49	67.25	66.99	66.09
	10-1074-LS (mg/mL)	76.70	78.78	77.93	77.34	76.60
**DLS**	Z-Average (d.nm)	10.25	10.49	10.72	11.07	12.06
	PDI	0.18	0.19	0.19	0.22	0.27
**FlowCAM**	2-10 µm (P/mL)	191	398	191	191	180
	10-25 µm (P/mL)	31	115	69	99	9
	25-50 µm (P/mL)	15	38	15	15	9

Table S6. Testing of functional activity of 3BNC117-LS and 10-1074-LS in the co-formulated samples from the 28-days stability study using pseudovirus neutralization assay. 2 weeks samples (T=2 weeks) and 4 weeks samples (T=4 weeks) were selected from all 3 conditions and analyzed against 2 panels of pseudoviruses in TZM-bl cells. One panel (**A and B**) is used to test functional activity of 3BNC117 [3BNC117 sensitive/10-1074 resistant viruses (n=10)] and the other panel (**C and D**) is used to test functional activity of 10-1074 [3BNC117 resistant/10-1074 sensitive viruses (n=10)]. Both IC50 and IC80 values (in µg/mL) are reported.

(A)

**Samples**	**ZM249M.PL1**		**Q461.e2**		**0013095-2.11**		**62357.14. D3.4589**		**ZM53M.PB12**	
**3BNC117.LS + 10-1074.LS**	**IC50**	**IC80**	**IC50**	**IC80**	**IC50**	**IC80**	**IC50**	**IC80**	**IC50**	**IC80**
T = 2 weeks (5 ± 3°C)	0.034	0.164	0.039	0.140	1.166	9.640	0.037	0.132	0.158	0.745
T = 4 weeks (5 ± 3°C)	0.032	0.123	0.031	0.116	0.826	18.425	0.030	0.142	0.166	0.647
T = 2 weeks (25 ±2°C/60%RH ± 5%)	0.040	0.142	0.045	0.161	1.064	14.495	0.039	0.135	0.198	0.710
T = 4 weeks (25 ±2°C/60%RH ± 5%)	0.034	0.123	0.032	0.123	0.956	12.547	0.019	0.095	0.147	0.597
T = 2 weeks (40 ±2°C/75%RH ± 5%)	0.047	0.164	0.044	0.159	1.325	16.869	0.051	0.174	0.190	0.672
T = 4 weeks (40 ±2°C/75%RH ± 5%)	0.035	0.127	0.033	0.129	0.847	20.476	0.027	0.128	0.186	0.704

(B)

**Samples**	**C2101.c01**		**C4118.c09**		**THRO4156.18**		**3415.v1.c1**		**CNE5**	
**3BNC117.LS + 10-1074.LS**	**IC50**	**IC80**	**IC50**	**IC80**	**IC50**	**IC80**	**IC50**	**IC80**	**IC50**	**IC80**
T=2 weeks (5 ± 3°C)	0.018	0.082	0.026	0.122	1.875	9.421	0.043	0.121	0.148	0.985
T = 4 weeks (5 ± 3°C)	0.033	0.123	0.042	0.156	1.493	8.361	0.040	0.157	0.147	0.706
T = 2 weeks (25 ±2°C/60%RH ± 5%)	0.020	0.092	0.035	0.168	1.879	6.913	0.047	0.128	0.181	0.828
T = 4 weeks (25 ±2°C/60%RH ± 5%)	0.023	0.108	0.030	0.143	1.641	6.068	0.047	0.140	0.137	0.907

T= 2 weeks (40 ±2°C/75%RH ± 5%)	0.034	0.158	0.048	0.219	1.754	6.625	0.049	0.132	0.190	0.897
T = 4 weeks (40 ±2°C/75%RH ± 5%)	0.029	0.133	0.040	0.186	2.108	7.518	0.055	0.168	0.183	0.875

(C)

Samples	1394C9_G1 (Rev-)		ZM247v1 (Rev-)		Du422.1		6631.v3.c10		377.v4.c9	
**3BNC117.LS + 10-1074.LS**	**IC50**	**IC80**	**IC50**	**IC80**	**IC50**	**IC80**	**IC50**	**IC80**	**IC50**	**IC80**
T=2 weeks (5 ± 3°C)	0.025	0.075	0.022	0.102	0.034	0.121	0.205	0.983	0.422	1.382
T= 4 weeks (5 ± 3°C)	0.026	0.081	0.028	0.103	0.031	0.119	0.197	0.993	0.416	1.432
T = 2 weeks (25 ±2°C/60%RH ± 5%)	0.023	0.084	0.027	0.122	0.043	0.122	0.209	1.004	0.487	1.252
T = 4 weeks (25 ±2°C/60%RH ± 5%)	0.024	0.094	0.025	0.094	0.029	0.108	0.176	0.872	0.402	1.129
T= 2 weeks (40 ±2°C/75%RH ± 5%)	0.021	0.075	0.028	0.129	0.045	0.126	0.186	0.909	0.451	1.202
T= 4 weeks (40 ±2°C/75%RH ± 5%)	0.027	0.101	0.032	0.148	0.049	0.171	0.226	1.041	0.395	1.093

(D)

Samples	20915593		T278-50		21197826-V1		Du151.2		19715820_A10_H2	
**3BNC117.LS + 10-1074.LS**	**IC50**	**IC80**	**IC50**	**IC80**	**IC50**	**IC80**	**IC50**	**IC80**	**IC50**	**IC80**
T=2 weeks (5 ± 3°C)	1.560	5.847	1.711	11.559	0.664	2.243	0.005	0.018	0.057	0.204
T=4 weeks (5 ± 3°C)	1.546	5.765	1.046	11.831	0.495	1.747	0.004	0.012	0.050	0.241
T = 2 weeks (25 ±2°C/60%RH ± 5%)	1.945	6.989	1.657	11.450	0.638	2.109	0.005	0.015	0.055	0.253
T= 2 weeks (40 ±2°C/75%RH ± 5%)	1.833	5.339	1.505	11.454	0.604	2.037	0.005	0.015	0.058	0.267
T =4 weeks (25 ±2°C/60%RH ± 5%)	1.704	6.266	2.047	14.448	0.555	1.937	0.005	0.015	0.063	0.230
T= 4 weeks (40 ±2°C/75%RH ± 5%)	1.828	5.308	2.358	15.848	0.667	2.238	0.006	0.018	0.075	0.262
